# Preparation of Protein Nanoparticles Using NTA End Functionalized Polystyrenes on the Interface of a Multi-Laminated Flow Formed in a Microchannel

**DOI:** 10.3390/mi8010010

**Published:** 2017-01-03

**Authors:** Hyeong Jin Jeon, Chae Yeon Lee, Moon Jeong Kim, Xuan Don Nguyen, Dong Hyeok Park, Hyung Hoon Kim, Jeung Sang Go, Hyun-jong Paik

**Affiliations:** 1 School of Mechanical Engineering, Pusan National University, 2 Busandaehak-ro 63beon-gil, Geumjeong-gu, Busan 46241, Korea; hjjeon@pusan.ac.kr (H.J.J.); mjkim80@pusan.ac.kr (M.J.K.); nguyenxuandon@pusan.ac.kr (X.D.N.); dhpark90@pusan.ac.kr (D.H.P.); 2 Department of Polymer Science, Pusan National University, 2 Busandaehak-ro 63beon-gil, Geumjeong-gu, Busan 46241, Korea; chaeyeonlee@pusan.ac.kr; 3 Boditech Med Inc., 43, Geodudanji 1-gil, Dongnae-myeon, Chuncheon-si, Gang-won-do 24398, Korea; khh@boditech.co.kr

**Keywords:** protein nanoparticles, microfluidic synthesis, self-assembly, lamination flow, protein-polymer hybrid

## Abstract

This paper challenges the production of the protein nanoparticles using the conjugation of Ni^2+^ complexed nitrilotriacetic acid end-functionalized polystyrene (Ni-NTA-PS) and histidine tagged GFP (His-GFP) hybrid. The microfluidic synthesis of the protein nanoparticle with the advantages of a uniform size, a fast reaction, and a precise control of preparation conditions is examined. The self-assembly occurs on the interfacial surface of the multi-laminated laminar flow stably formed in the microchannel. The clogging of the produced protein nanoparticles on the channel surface is solved by adding a retarding inlet channel. The size and shape of the produced protein nanoparticles are measured by the analysis of transmission electron microscopy (TEM) and scanning electron microscope (SEM) images, and the attachment of the protein is visualized with a green fluorescent image. Future research includes the encapsulation of vaccines and the coating of antigens on the protein surface.

## 1. Introduction

The development of nanoparticles has been rapidly increased to deliver various therapeutic and diagnostic agents such as molecules, proteins, and peptides with the distinct advantages of a precise control of the size, morphology, and surface functionalization for potential use in drug delivery and pharmaceutics [1,2,3,4,5]. They are colloidal structures of nanometer size and are designed to control the release of the entrapped molecules over time at an optimal rate to maintain drug concentrations at a specific site of treatment.

A variety of materials have been explored to prepare the nanoparticles by considering the targeted size, the affinity to the encapsulated drugs and molecules, the release profile, biocompatibility, biodegradability, and the toxicity of nanoparticles. Recent developments in pharmaceutical applications have paid more attention to protein nanoparticles due to their low toxicity and safety, improved therapeutic efficacy, and biodegradability compared with synthetic polymer nanoparticles [6,7,8].

Few protein nanoparticles have been synthesized from the water-soluble and -insoluble proteins such as albumin, gelatin, elastin, gliadin, and natural proteins. The proteins are ideal materials to synthesize nanoparticles because of their amphiphilic property, which provides excellent interaction with both the drug and the solvent [9,10,11,12,13,14,15,16]. Coacervation and emulsion methods are most commonly used for their preparation. In the coacervation method, the solubility of the protein is reduced, and the protein is then aggregated. By adding a cross linking agent, the aggregated proteins are changed into the protein structure, resulting in the coacervation of the protein [17,18,19,20]. The emulsion method forms nano-sized droplets of an aqueous solution of the protein in oil by using a high speed homogenizer, and the nanoparticles are formed on the interface of the aqueous solution droplets with oil [21,22,23].

Most preparation methods of the protein nanoparticles require sophisticated control of process parameters in order to produce the designed features of the nanoparticles and are bench-top batch processes for large quantity production. They typically have severe limitations in the controlled preparation of homogeneous and monodispersed nanoparticles and produce broadly distributed nanoparticles in size [24,25]. 

To these ends, microfluidic preparation methods have been reported recently with the benefit of a continuous synthesis of homogeneous polymer nanoparticles in a stable and controllable manner using a stable laminar fluid flow [26,27]. This method utilizes hydrodynamic focusing in a microchannel to drive by a self-assembly process. Two miscible fluids are introduced into the channel, a strong laminar flow is formed due to a low Reynolds number (*Re* < 0.1) resulting from the small hydraulic diameter (*D*_h_) defined by 4*A*/*P*, where *A* is the cross-sectional area of the microchannel, and *P* is the perimeter of the area. The laminated interface is strongly resistant to external disturbance hydrodynamically and the width of the laminated stream is simply controlled by changing the ratio of two inlet flow rates. This flow focusing synthesis in the microchannel can provide a stable and controllable synthesis of nanoparticles such as poly(lactic-co-glycolic acid) (PLGA) nanoparticles, liposomes, and quantum dots [28,29]. However, the direct application of the reported design of the microfluidic methods causes clogging problems in the microchannel with explosive reactions.

In this paper, we challenge the continuous and flow-through production of protein nanoparticles by using the microfluidic preparation and develop the microfluidic process to improve microchannel clogging occurring during the synthesis of the protein nanoparticles. In specific, polymer-protein composite nanoparticles are synthesized continuously using the self-assembly on the interface of the laminated flow. Moreover, the controllability in size and the morphology of the protein nanoparticles are examined experimentally.

## 2. Synthesis of Materials

**Figure 1:**
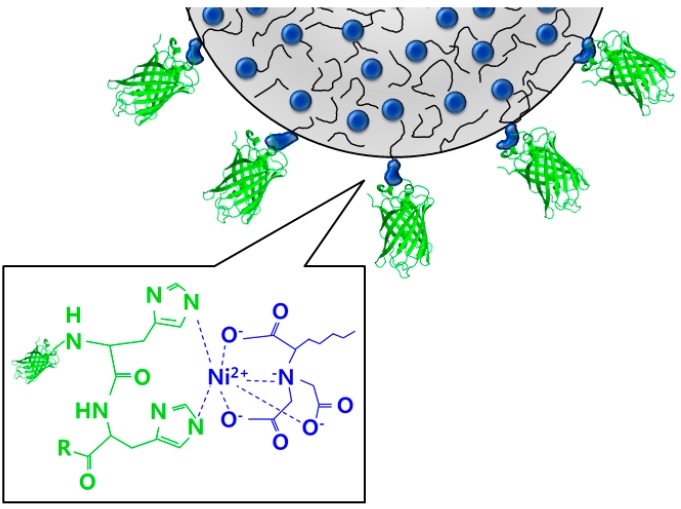
Configuration of the conjugation of the polymer and protein hybrid.

The complexation of polystyrene and protein is used for the synthesis of the protein nanoparticles [30,31,32]. Figure 1 shows the conjugation of polymer containing nitrilotriacetic acid end-functionalized polystyrene (NTA-PS) and histidine-tagged green fluorescence protein (His-GFP). It uses the non-covalent binding of the PS containing Ni^2+^ complexed nitrilotriacetic acid (Ni-NTA) to conjugate with His-GFP. The NTA occupies four of the six binding sites of Ni^2+^ and leaves two free sites for His-GFP. The Ni^2+^ complexation with the histidine is fast and reversible. Imidazole can displace the His-tag proteins [30,32].

The Ni^2+^ complexed nitrilotriacetic acid polystyrene (Ni-NTA-PS) is prepared via atom transfer radical polymerization (ATRP). Firstly, polystyrene of 300 mg and 61.2 × 10^−3^ mmol and trifuoroacetic acid of 300 μL and 18.6 × 10^−1^ mmol were stirred in CH_2_Cl_2_ of 20.0 mL at room temperature for 12 h for the deprotection of the *tert*-butyl group. The solvent was evaporated. The product was purified by using precipitation against MeOH and dried at 45 °C for 12 h. Secondly, the polystyrene of 100 mg and 61.2 × 10^−3^ mmol was dissolved in 50 mL of dimethylformamide (DMF) and then 54.4 mg of 0.42 mmol nickel chloride (NiCl_2_) was added. The complexation was conducted by stirring at room temperature for 12 h. The mixture was precipitated to purify against MeOH and dried at 45 °C for 12 h. Finally, a mixture of 2 mg was dissolved in 2 mL of THF, and 8 mL of water was slowly added while stirring. The THF was removed by continuous stirring for 3 h and at a temperature of 45 °C. The resulting solution was sonicated for 30 min and kept at 4 °C. 

His-GFP was expressed from cells. *Escherichia coli* BL21 (DE3) harboring the pET-GFPmut3.1 was grown to express GFPmut3.1 tagged with hexahistidine at its N-terminus in a medium of 100 mL of Luria-Bertani (LB) containing 100 μg/mL ampicillin and was induced with isopropyl 0.05 mM β-D-thiogalactopyranoside for 6 h at 37 °C, and then pelleted. By using protein extraction kit (BugBuster, Novagen, Merck Millipore, Darmstadt, Germany), cells were lysed. The collected cell pellets were resuspended in a 5 mL lysis buffer and incubated at room temperature for 10 min. Then, they were centrifuged for 20 min at 9000 g at 4 °C. The extracts were bound to 5 mg of resin (Ni-NTA-His∙Bind Resin, Novagen) after incubation for 3 h at 4 °C, and the resin was loaded into the column. It was washed with a 4 mL washing buffer consisting of 50 mM phosphate buffer with a pH of 8, 300 mM NaCl, and 20 mM imidazole. The His-GFP was eluted with an elution buffer of 1 mL consisting of 50 mM phosphate buffer with a pH of 8, 300 mM NaCl, and 250 mM imidazole. The imidazole in the eluted solution was removed using centrifugal filtration (Ultra Diafiltration, EMD Millipore, Merck Millipore). The protein fractions were separated using the polyacrylamide gel electrophoresis sodium dodecyl sulfate (SDS-PAGE) and the purity of the His-GFP was more than 95% [30,31,32].

## 3. Microfluidic Preparation of Protein Nanoparticles

In a conventional preparation procedure, the Ni-NTA-PS (*M*_n_ = 21,800) dissolved in DMF and a His-GFP PBS buffer of pH 7.4 are injected slowly into deionized water by using a syringe pump and stirred at 250 rpm for 5 h at 25 °C. Then, they start to aggregate spherically by self-assembly. Additionally, it is nonspecific irreversible binding because the conjugation and release of His-GFP from Ni-NTA-PS can be controlled via imidazole treatment. However, in the conventional method, this process takes five hours, and the reaction conditions must be controlled in a sophisticated manner. In addition, the dispersity in the conventional preparation was reported to be 17%–45%, which indicates a difficulty in the control of the particle size [33].

To these ends, we examined the microfluidic synthesis with the advantages of the control of uniform size, fast reaction, and a precise control of the reaction conditions for the preparation of the protein nanoparticles. When two miscible fluids were introduced into microchannel, the fluids were laminated because the viscous force was more dominant than the inertial force in the microchannel with a small hydraulic diameter [28,34]. The self-assembly on the interface of the laminated flow indicates a precise reaction time by manipulating the inlet flow rates for the constant amount and concentration of polymers. Additionally, the microscale diameter of the microchannel has a short heat transfer distance, resulting in a small distribution of temperature so that the temperature of the reaction condition can be kept constant [35,36,37].

To fabricate the microfluidic platform, we used poly-dimethylsiloxance (PDMS) mold bonded with a transparent slide glass. A negative SU-8 photoresist (SU-8 2050, MicroChem, Westborough, MA, USA) was spin-coated onto a silicon wafer. The solvent was removed by heating to 65 °C for 3 min, followed by a further 9 min curing at 95 °C to increase film density. The SU-8 photoresist was exposed to UV with a wavelength of 365 nm for 25 s, and the photoresist was developed in SU-8 developer. Then, the patterned SU-8 mold was dried in an oven at 110 °C after cleaning. The width and height of the fabricated SU-8 mold were measured to be 50 and 80 μm, respectively, by using a non-contact surface profiler (ET 400Am, Kosaka Lab Ltd., Tokyo, Japan). The downstream length after the focusing section was 3 mm.

Copper ports were bonded over the SU-8 mold to form inlets and outlets. After the PDMS resin was poured onto the SU-8 mold, air bubbles were perfectly removed by vacuum pump. Then, it was cured at 110 °C for 30 min. Finally, the patterned PDMS was detached from the SU-8 mold. Oxygen plasma (PDC-32G, Harrick Plasma) was used to modify the surface property of the transparent glass slide and the PDMS mold for their bonding. Following oxygen plasma treatment, the surfaces were brought into immediate contact to form the microchannel. Finally, the channel surface was modified hydrophobically with a self-assembled monolayer of dichlorodimethylsilane using liquid phase vaporization. 

**Figure 2:**
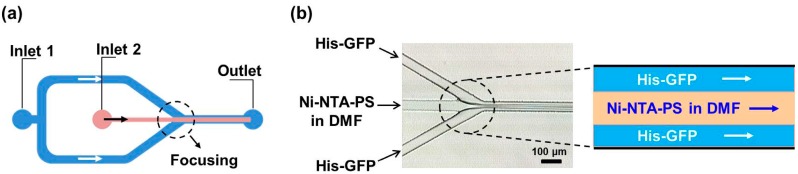
Schematic of the flow-focusing channel: (**a**) design of the flow-focusing channel; (**b**) the three-layered lamination flow. His-GFP: histidine tagged GFP; Ni-NTA-PS: nitrilotriacetic acid end-functionalized polystyrene; DMF: dimethylformamide.

Figure 2a shows the most commonly used design of the microfluidic preparation of nanoparticles. It consists of a central inlet channel and two side inlet channels and an outlet. The Ni-NTA-PS solution was introduced into the center inlet channel with a flow rate of 40 μL/min and the His-GFP PBS buffer into two side inlet channels with a flow rate of 20 μL/min. Additionally, the Reynolds number was calculated to be 3.45 for the His-GFP solution and 6.87 for the polymer solution, respectively, which indicates that the flow is in a strong laminar flow regime. As visualized in Figure 2b, a distinct lamination flow was formed.

**Figure 3:**
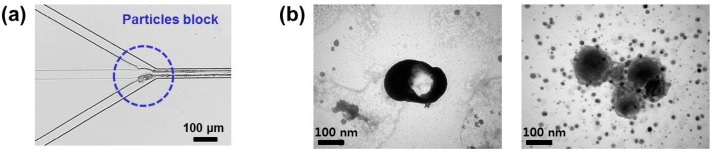
Problem of the flow-focusing synthesis: (**a**) aggregation and clogging of the protein nanoparticles at the junction of inlet channels and along the channel; (**b**) transmission electron microscopy (TEM) images of the aggregated particles.

However, due to explosive polymerization between Ni-NTA-PS and His-GFP, the protein nanoparticles aggregated at the junction of the inlet channels as shown in Figure 3a, and the aggregation occurred along the microchannel to the outlet channel. The aggregated nanoparticles were attached on the channel surface and they disturbed the lamination flow. As a result, this caused the aggregated and uncontrolled protein nanoparticles to be collected from the outlet channel. Figure 3b shows the transmission electron microscopy (TEM, Hitachi, Tokyo, Japan) images of the collected protein nanoparticles.

**Figure 4:**
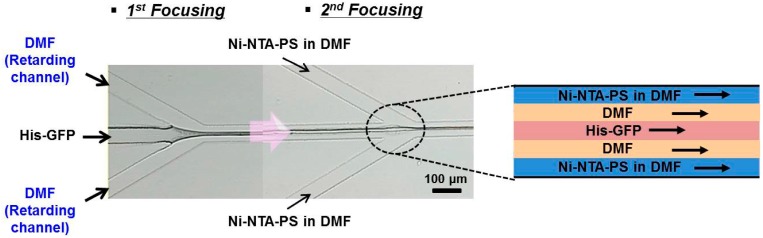
Multi-layered lamination flow formed with two-step focusing channel.

In order to improve the problem of aggregation, we examined the addition of a retarding inlet channel into the flow-focusing synthesis channel. This retarding channel delays the direct contact of Ni-NTA-PS, His-GFP, and their self-assembly. As shown in Figure 4, the His-GFP solution is injected through the central inlet channel. Pure DMF without Ni-NTA-PS is introduced into the retarding inlet channel. At the first junction of the two inlet channels, the three-layered lamination flow forms. Owing to the amphiphilic property of the His-GFP, they arrange on the interface. The DMF solution with Ni-NTA-PS is injected through the side inlet channels. Then, the width of the three-layered flow formed formerly is reduced by the flow of the Ni-NTA-PS solution at the second junction. Finally, the five-layered flow forms.

The width of the pure DMF layer can be regulated by controlling both of the inlet flow rate of the retarding inlet channel and that of the side inlet channels. The Ni-NTA-PS diffuses to meet the arranged His-GFP and they are self-assembled. Therefore, the position of their self-assembly can be controlled near the outlet channel. As a result, this improves the aggregation in the synthesis microchannel. 

Figure 4 visualizes the formation of the five-layered lamination flow. To the central inlet channel, the buffer solution with His-GFP PBS was introduced with a flow rate of 125 μL/min. The pure DMF was injected with a flow rate of 40 μL/min into the retarding inlet channel. Additionally, the Ni-NTA-PS dissolved in DMF was introduced through the side inlet channels with a flow rate of 10 μL/min. At the second junction, only a three-layered lamination with a width of about 10 μm each was observed because the retarding layer had the same DMF with the Ni-NTA-PS dissolved in DMF, but it should have been a five-layered lamination flow. Additionally, it was shown that the width of the first three laminated layers was decreased by the flow of the Ni-NTA-PS solution. The protein nanoparticles were formed in the interface of the laminated flow and the residence time for self-assembly was calculated to be 10.68 ms from the length between the second focusing junction to the outlet channel. Compared with conventional methods, the microfluidic synthesis can provide a fast reaction for the preparation of the protein nanoparticle. 

**Figure 5:**
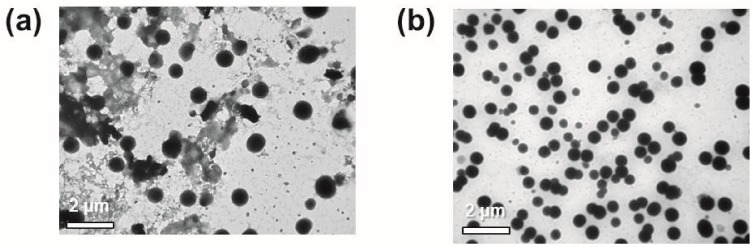
A comparison of TEM images with different focusing steps: (**a**) flow focusing without the retarding inlet channel; (**b**) flow focusing with the retarding inlet channel.

Figure 5 compares the TEM images of the protein nanoparticles collected from the flow focusing microfluidic synthesis channel without and with the retarding inlet channel. This comparison confirms that the retarding inlet channel can reduce the remaining GFP wastes and make a higher preparation yield of the protein nanoparticles. 

## 4. Evaluation of the Produced Protein Nanoparticles

**Figure 6:**
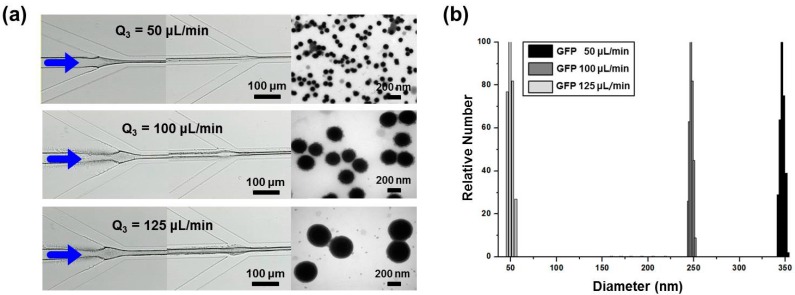
Measurement of the produced protein nanoparticles with different flow rates of GFP: (**a**) TEM images of the controlled particle size; (**b**) dynamic light scattering (DLS) measurement.

To examine the size control of the protein nanoparticles, the inlet flow rate of the His-GFP PBS were adjusted 50, 100, and 125 μL/min, respectively. The inlet flow rate of the Ni-NTA-PS DMF was fixed to be 10 μL/min. Especially, Figure 6 shows the TEM images and measurement results of dynamic light scattering (DLS). The size of the protein nanoparticles also increased to 49, 237, and 350 nm, respectively, as the inlet flow rate of the His-GFP PBS buffer increased. The reason for the increase in size can be explained by the fact that the increase of the inlet flow rate of the His-GFP at the central inlet channel reduces the width of the pure DMF introduced from the retarding inlet channel layer. As a result, the size of the protein nanoparticles can be increased. The size control of the protein nanoparticles is summarized in Table 1.

**Figure 7:**
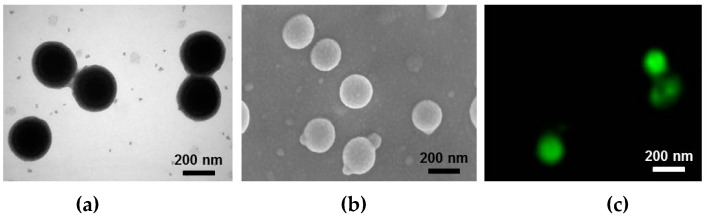
Evaluation of the produced protein nanoparticles: (**a**) TEM image; (**b**) scanning electron microscope (SEM) image; (**c**) confocal fluorescence image.

Figure 7 evaluates the produced protein nanoparticles. Their shape and size were examined by using TEM and field emission scanning electron microscope (FESEM, Zeiss, Jena, Germany). The uniform size of the protein nanoparticles was measured to be 250 nm with a small size distribution and they have a spherical shape. Additionally, the green fluorescent image taken from a super resolution confocal microscope (TCS SP8-gSTED, Leica, Wetzlar, Germany) confirms the attachment of the proteins on the surface of polystyrenes. This protein surface can be applied to biomedical and pharmaceutical fields. 

## 5. Conclusions

This paper examines the fast and controllable preparation of the protein nanoparticles in the flow-focusing synthesis channel. The Ni-NTA-PS and His-GFP was self-assembled on the interfacial surface of the multi-laminated flow. Especially, the problem of aggregation and clogging of the self-assembled protein nanoparticles on the inner surface of the channel was improved by adding the retarding inlet channel. The width of the solution introduced from the retarding inlet channel could be controlled simply by regulating the inlet flow rates of the central inlet channel and the retarding inlet channel, which indicates the possible control of the duration of the self-assembly. 

It is demonstrated that the microfluidic synthesis has the advantages of a uniform size control, a fast reaction, and a precise control of the reaction conditions for the preparation of protein nanoparticles by regulating inlet flow rates. For future study, the attachment of various antigenic molecules and the encapsulation of drugs inside the protein nanoparticles can be challenged.

## Figures and Tables

**Table 1:** Size control of the protein nanoparticles by changing the flow rate of the His-GFP solution.

Entry	Flow Rate of His-GFP (μL/min)	Lamination Width of Second Focusing (μm)	Mean Diameter of Nanoparticle (nm)
1	50	8.06	49 ± 3
2	100	11.29	237 ± 13
3	125	12.90	350 ± 16
